# Hypernatremia in an Infant: A Case of Septo-Optic Dysplasia

**DOI:** 10.7759/cureus.12450

**Published:** 2021-01-03

**Authors:** Oluwatosin O Oyadiran, Naxdaris Gonzalez, Ahmad Khiami

**Affiliations:** 1 Pediatrics, Raleigh General Hospital, Beckley, USA; 2 Pediatrics, Beckley Appalachian Regional Healthcare, Beckley, USA; 3 Pediatrics, West Virginia University, Morgantown, USA

**Keywords:** septo-optic dysplasia, sod, optic nerve hypoplasia, onh, absent septum pellucidum, panhypopituitarism, diabetes insipidus, hypernatremia, dehydration

## Abstract

Untreated and rapid correction of neonatal hypernatremia leads to severe neurological complications. We describe the case of a six-week-old female who presented with failure to thrive, and further workup revealed hypernatremic dehydration. Initially, she did not respond to treatment to correct the hyperosmotic state. Treatment with desmopressin was then initiated to determine the cause of hypernatremia. Central diabetes insipidus was confirmed as the patient responded to desmopressin. Serum sodium levels then dropped significantly, and the patient had three seizures within 24 hours. Cerebral edema was ruled out through computed tomography (CT) and electroencephalogram. Following the diagnosis of central diabetes insipidus, anterior pituitary hormone levels were obtained and found to be decreased. An investigation into the possible cause of panhypopituitarism led to the final diagnosis of septo-optic dysplasia, including absent septum pellucidum, optic nerve hypoplasia, and panhypopituitarism.

## Introduction

Septo-optic dysplasia (SOD) or De Morsier syndrome is a multifarious disorder involving at least two of the following anomalies: midline brain abnormalities, optic nerve hypoplasia (ONH), and hypothalamic-pituitary dysfunction [1]. It has been associated with HESX1, OTX2, and SOX2&3 gene mutations, and environmental influences during organogenesis [2,3]. It has also been linked to in utero injuries (e.g. viral infections, vascular disruption of the anterior cerebral artery, or maternal exposure to medications) [2]. Late interventions can be fatal, with early signs varying between hypoglycemia, developmental delay, adrenal crisis, seizures, and death [4,5]. The case being discussed is of a female who presented with failure to thrive at infancy.

## Case presentation

A six-week-old female infant presented for follow-up on failure to thrive. The patient was born vaginally at term. Initial assessment at birth included Apgar scores of 8 and 9 at one and five minutes, respectively. Newborn screening tests were normal, and the patient was visibly jaundiced. Laboratory tests revealed hypoglycemia and leukocytosis. The patient was initially managed with phototherapy and empiric antibiotics, but antibiotic therapy was discontinued following negative cultures. She remained jaundiced during her first well-child visit in the first week. She had failed to gain weight during her one-month wellness check, and her mother was advised to increase feeding and follow-up in two weeks. On presentation at six weeks, the patient's weight was 6.12 lb compared to a birth weight of 6.13 lb and a weight of 6.6 lb during hospital discharge (six days post-delivery). Bowel movement and diet (three ounces every three hours of Similac Sensitive® supplemented with breast milk) were regular, with no gastrointestinal complaints. She remained jaundiced with icteric sclera. The rest of the examination was insignificant, and the patient was admitted to monitor diet. Laboratory studies on admission showed hypernatremia (154 mmol/L), hyperbilirubinemia (total bilirubin 7.6 mg/dL), lymphocytosis, and increased alkaline phosphatase (Tables 1, 2). These findings confirmed hypernatremic dehydration. Intravenous fluid replacement therapy (D5 quarter normal saline) was immediately initiated to correct extracellular fluid depletion. The patient's sodium levels did not improve in the following days, and the patient was transferred to a specialist hospital. In the absence of hyperglycemia (ruling out osmotic diuresis due to diabetes mellitus) and poorly reconstituted formula, the following diagnoses of central diabetes insipidus and nephrogenic diabetes insipidus were made.

**Table 1:** Complete Blood Count Showing Increased RDW, Lymphocytosis, and Eosinophilia WBC, white blood cells; RBD, red blood cells; Hb, hemoglobin; Hct, hematocrit; MCV, mean corpuscular volume; MCH, mean corpuscular hemoglobin; MCHC, mean corpuscular hemoglobin concentration; RDW, red cell distribution width

Test	Initial admission		Second admission (specialist hospital)		Reference	Unit
	Day 1	Day 2	Day 1	Day 2		
WBC	12.1	10.8	14.3	10.2	6.2-10.9	k/mm^3^
RBC	4.17	3.53	3.63	2.94	3.10-5.0	mil/mm
Hb	12.7	10.6	11.3	9.0	9.0-13.4	g/dL
Hct	37.1	30.9	34.3	27.2	37.8-66.0	%
MCV	89	88	94	93	82.0-88.0	um^3^
MCH	31	30	31.1	30.6	28.0-30.0	pg
MCHC	34	34	33.0	33.1	33.5-34.0	%
RDW	16.4	16.5	15.9	16.3	11.5-14.0	%
Platelets	318	354	279	291	140-450	k/mm^3^
Segmented neutrophils	10	10	25	16	17-71	%
Lymphocytes	77	76	66	74	23-58	%
Monocytes	6	8	6	9	5-15	%
Eosinophils	7	6	3	1	0-2	%
Poikilocytosis	Slight	1+	Slight	Slight	Negative	
Anisocytosis	-	1+	Moderate	Slight	Negative	
Target cells	-	1+	Slight	Slight	Negative	

**Table 2:** Complete Metabolic Panel (on Initial Admission) Showing Hypernatremia, Hyperbilirubinemia, and Elevated Alkaline Phosphatase Levels BUN, blood urea nitrogen; AST, aspartate transaminase; ALT, alanine transaminase

Test	Initial admission		Reference	Unit
	Day 1	Day 2		
Sodium	154	158	137-144	mmol/L
Potassium	5.3	5.0	3.5-5.0	mmol/L
Chloride	122	127	98-108	mmol/L
CO_2_	20	20	21-32	mmol/L
Glucose	143	60	55-115	mg/dL
BUN	21	15	7-18	mg/dL
Creatinine	0.3	0.4	0.6-1.1	mg/dL
Calcium	10.9	10.2	8.5-10.1	mg/dL
Total bilirubin	7.6	6.2	0.0-5.0	mg/dL
AST	46	32	15-37	units/L
ALT	22	17	12-78	units/L
Alkaline phosphatase	522	517	82-383	units/L

**Figure 1:**
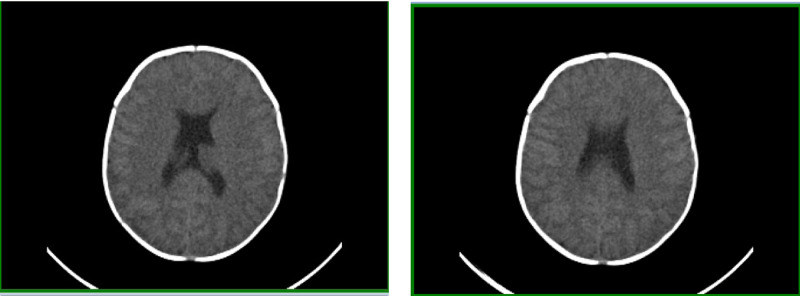
Computed Axial Tomography Ruling Out Cerebral Edema

On admission, the patient was started on 0.1 mcg of desmopressin every 12 hours on the condition that serum sodium was above 135 mmol/L. Serum sodium levels were checked one hour before administering desmopressin. The patient responded well as serum osmolarity decreased and urine osmolality increased. The constellation of symptoms, including hypernatremia, low urine osmolarity, low specific gravity, and the improvements after administration of desmopressin, confirmed central diabetes insipidus. Later that day, she suffered from three episodes of generalized seizures. The first seizure was the longest, lasting for four minutes, while the other episodes lasted for two minutes each. The patient was afebrile during the first two episodes, but she felt warm to touch during the third seizure; however, no formal reading was taken. Laboratory values showed that serum sodium levels had dropped from 159 mmol/L to 137 mmol/L within the last 24 hours (Table 3). She was transferred to the pediatric ICU, treated with levetiracetam, and managed empirically with ampicillin, cefotaxime, and acyclovir for meningoencephalitis. Lumbar puncture, computed tomography (CT) (Figure 1), and electroencephalogram were performed to rule out suspected meningoencephalitis and other causes of seizures. All tests turned up unremarkable, and no epileptiform abnormalities were seen. Despite normal brain activity, management with levetiracetam was continued throughout admission.

**Table 3:** Basal Metabolic Panel (on the Second Admission) Showing a Rapid Reduction of Sodium Within the First 24 Hours

Test	Second admission				Reference	Unit
	Day 1	Day 2, A.M.	Day 2, P.M.	Day 7		
Sodium	159	146	137	140	137-144	mmol/L
Potassium	4.4	5.0	5.6	5.1	3.5-5.0	mmol/L
Chloride	123	110	103	104	98-108	mmol/L
CO_2_	23	23	24	24	21-32	mmol/L
Glucose	82	75	85	80	55-115	mg/dL
Blood urea nitrogen	7	6	7	7	7-18	mg/dL
Creatinine	0.1	0.2	0.2	0.2	0.6-1.1	mg/dL
Calcium	9.8	9.8	9.4	9.6	8.5-10.1	mg/dL

**Figure 2:**
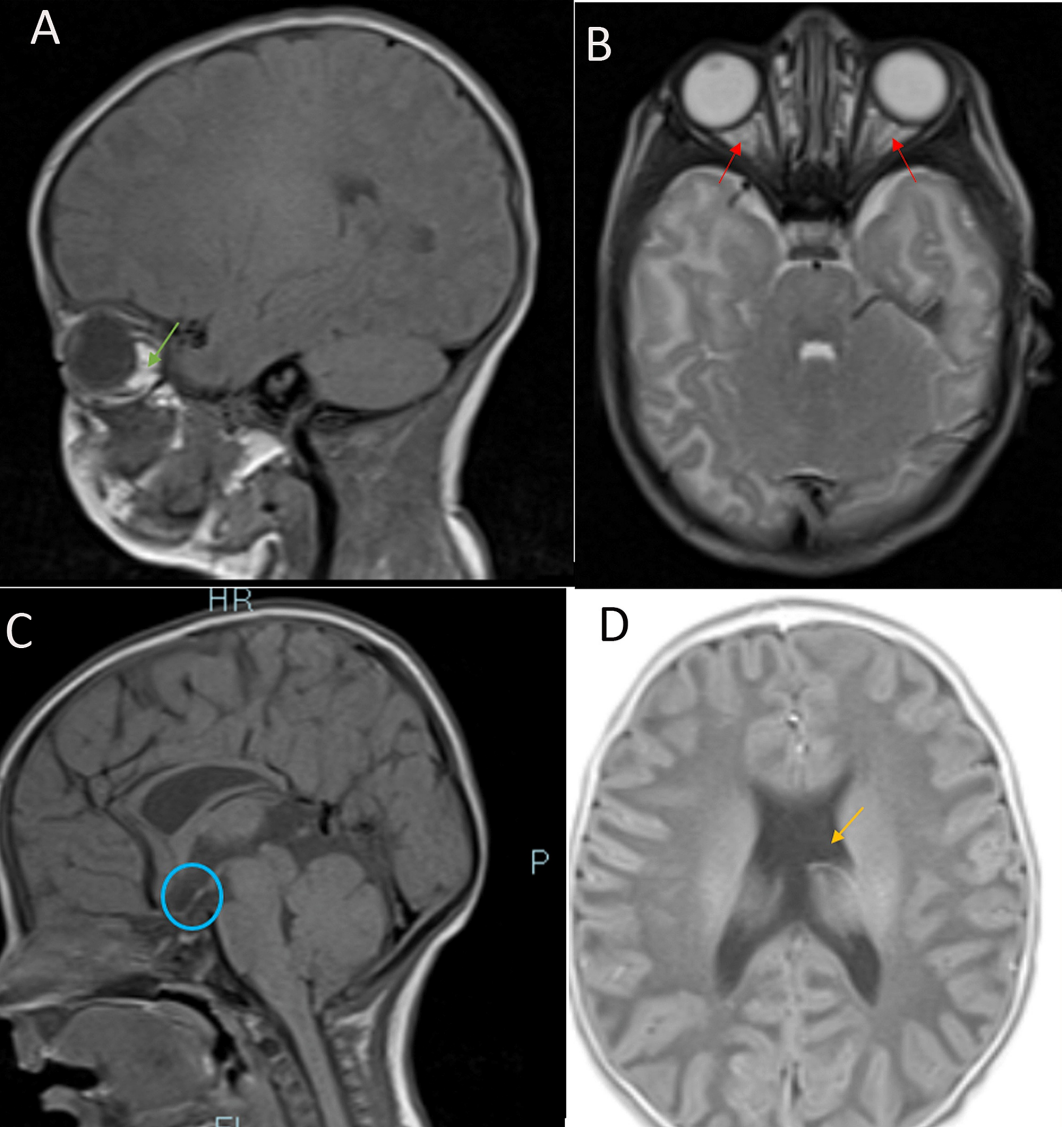
MRI of the Brain and Pituitary. (A) T1-weighted sagittal view. Hypoplasia of the optic nerve (green arrow). (B) T2-weighted axial view. Hypoplasia of the right and left optic nerves (red arrows). (C) T1-weighted, sagittal view. Hypoplastic pituitary stalk (circled in blue). (D) T2-weighted axial view. Complete absence of the septum pellucidum (seen as mono-ventricle in orange arrow).

Anterior pituitary hormones baseline values were obtained, and cortisol, thyroid, and growth hormone levels were decreased (Table 4). The patient also experienced episodes of hypoglycemia throughout her hospital stay. Following the confirmation of central diabetes insipidus and reduced anterior pituitary hormones, the diagnosis was made for panhypopituitarism, and magnetic resonance imaging (MRI) of the brain and pituitary were ordered. MRI revealed hypoplasia of the pituitary stalk, an absent septum pellucidum, and suspected hypoplasia of the optic nerve (Figure 2), confirming SOD. Ophthalmologic consult reported that the pupils were not reactive to light and fundoscopy confirmed hypoplastic optic discs bilaterally.

**Table 4:** Anterior Pituitary Hormone Tests Showing Panhypopituitarism TSH, thyroid-stimulating hormone; T4, thyroxine; GH, growth hormone; FSH, follicle-stimulating hormone; LH, luteinizing hormone; IGF, insulin-like growth factor

Test	Results	Reference	Unit
TSH	5.46	0.358-3.740	uIU/mL
T4	0.87	0.76-1.46	ng/dL
GH	2.8	0.0-10.0	ng/mL
Cortrosyn 30m	16.8		
Cortrosyn 60m	15.5		
FSH	0.174	1-4.2	ng/dL
LH	0.01	0.02-7	mIU/mL
IGF	55	15-109	ng/mL
IGFβ	0.3	0.5-2.1	mg/L

## Discussion

Hypernatremia

Hypernatremia is defined as serum sodium levels greater than 150 mmol/L. It may be caused by the loss of total body water or decreased fluid intake. Loss of total body water (hypotonic loss) occurs commonly with childhood diarrhea from rotavirus infection [6]. Common examples of decreased fluid intake in infants include improperly prepared oral rehydration salt (ORS) solution, inappropriately reconstituted infant formula, and insufficient breastfeeding [7,8]. Hypernatremia is also common in critically ill patients, and it has been hypothesized to be a part of the body's defense to inflammation [9]. Acute complications of elevated sodium levels include renal failure and neurologic symptoms, and chronic hyperosmolar states may cause developmental delay in children [10,11]. Neurologic presentations include reduced or loss of consciousness, irritability, insomnia, cerebral edema, subdural effusion, intracerebral hemorrhage, seizures, cavernous sinus thrombosis, and coma. Patients may also present with weight loss, poor feeding, hyperthermia, restlessness, lethargy, mucosal dryness, jaundice, apnea, and cyanosis [6,7,10]. Diagnosis is made by assessing plasma and urinary sodium levels, along with urinary and plasma osmolarity.

Ideally, the body prevents hypernatremia through increased thirst and water retention (through vasopressin release). The patient in our case suffers from panhypopituitarism and, therefore, cannot produce vasopressin or concentrate urine in response to hyperosmotic states. Acute changes to the brain in response to hypernatremia include cerebral contraction following fluid loss. The brain adapts by absorbing water from the cerebrospinal fluid and releasing solute into the cells. After this adaptation, reverting chronic hypernatremia too quickly may cause cerebral edema [7]. It is therefore important to slowly correct sodium levels in these patients. This can be done by ensuring an hourly reduction by less than 0.5 mEq/L (12 mEq/L/day). It is important to monitor closely as a study showed that patients with a reduction of 0.7 mEq/L/hr or more developed complications [7]. Carefully administration of enteral fluids or hypotonic saline with 5% dextrose ideally corrects extracellular fluid depletion. Isotonic saline worsens hypernatremia and should only be administered if hemodynamically unstable and replaced with hypotonic saline once stable. A study successfully treated patients with >200 mmol/L of serum sodium using ORS, citing the challenge of the inability to maintain acceptable daily loss [6]. This patient's hypernatremic state could not be initially managed due to an underlying diabetes insipidus. She responded to treatment following desmopressin administration.

Despite the careful reduction of serum sodium levels, there was a significant drop of 22 mEq/L within 24 hours. The cause of the sudden drop is unknown, but this may have triggered the seizures our patient had. The seizures could have also been due to hypernatremic complications or symptoms of SOD. The patient did not have any more seizures, and therefore it is more likely that the rapid sodium drop caused the seizures. CT did not show cerebral edema; hence, there was no indication for hypertonic saline. Throughout her hospital stay, levetiracetam was used for seizure prophylaxis. It was subsequently discontinued as there was no indication for use after discharge.

Septo-optic dysplasia

Patients with SOD may present at birth (congenital adrenal hyperplasia, congenital hypothyroidism) or in the first few years of life (e.g. failure to thrive, seizures, hypoglycemia, hypernatremia, developmental delay, bilateral visual impairment). They may also present with delayed puberty in adolescents, endocrine insufficiency in adulthood, or treatment-resistant seizures at any age [12]. Our patient presented with absent septum pellucidum, panhypopituitarism, and ONH.

Midline brain abnormalities in SOD may present as either dysgenesis (or absence) of corpus callosum, septum pellucidum, or both. It can also present as hypoplasia of the falx or fornix [2,4]. Antenatal transabdominal and transvaginal ultrasound is vital in determining the flow of cerebrospinal fluid, the position of the choroid plexus, cleavage of the hemispheres, and deep gray nuclei and callosal defects in suspected SOD [13]. Prenatal ultrasound in this patient, however, showed no anomalies. SOD patients with midline brain defects may present with other cerebral malformations. A study revealed an association of SOD with pachygyria, polymicrogyria, and schizencephaly [4]. Another study of a preterm African male with SOD showed absent septum pellucidum and dilated third ventricle on cranial ultrasound, with MRI of the brain revealing thin corpus callosum, bilateral blunted roof of the lateral ventricles, and pachygyria [4]. The patient in the study presented with absent septum pellucidum confirmed by cranial MRI postnatally. Cases of absent septum pellucidum are uncommon; however, it may occur alone or in association with SOD, hydrocephalus, encephalocele, holoprosencephaly, porencephaly, and hydranencephaly [13].

The presentation of hypothalamic-pituitary dysfunction in SOD ranges from unexplainable hormone(s) deficiency to hypoplastic or absent pituitary stalk. The most common hormone deficiencies associated with SOD are growth and thyroid hormones, and these patients may experience developmental delay [14]. There is also a risk of precocious or late puberty, with the latter occurring in patients not receiving growth hormone supplementation [15]. Although newborn screening for this patient was negative for CAH and congenital hypothyroidism, it is very useful in diagnosing endocrinopathies that present at birth. Since hospitalization, our patient has been followed regularly by an endocrinologist. She has been placed on desmopressin and replacement therapy for cortisol, thyroid, and growth (somatotropin) hormones. Bone age was three years and six months at the chronological age of five years and eight months, indicating delayed skeletal maturation.

Clinical manifestations of ONH include microphthalmia, unilateral or bilateral color blindness, diminished visual acuity, coloboma, astigmatism, refractive errors, and complete blindness [1,12]. A fetal brain MRI or triplanar neurosonography is useful for probing possible optic nerve involvement if SOD is suspected in utero [13]. Cranial MRI is also indicated in patients with ONH to rule out pituitary and brain midline malformations. Ophthalmoscopic examination in our patient showed hypoplastic optic discs in both eyes, and further evaluation confirmed complete blindness.

We interviewed the patient's mother in a bid to postulate possible events that could have led to SOD in this patient. Per mother's recollection, pregnancy was uneventful except for group B *Streptococcus* infection. The mother is non-diabetic and denied a history of gestational diabetes. We also cannot rule out genetic mutations in this case.

The patient is now six years old, and she has been growing over the years despite falling behind normal height and weight percentiles for her age. Her height increased progressively from birth but dropped by two growth curves at 27 months. There was a slow but steady increase in weight in the first six months, with a decline to <5th percentile at 27 and 30 months. Head circumference was less than 50th percentile in the first three years. She has not had any episodes of hypernatremia or dehydration since hospitalization. Social development has been satisfactory despite delayed milestones, and she interacts well at home, school, and social gatherings. Although her speech at age 3 was comparable to that of a 12- to 15-month toddler, her speech and language have slowly improved, and she has not shown any neurologic deficit or mental retardation.

## Conclusions

Our patient was admitted for failure to thrive, and extensive workup led to the diagnosis of SOD. Early detection of infantile hypernatremia is important to prevent serious complications that could be lethal to infants. It is imperative to slowly correct sodium levels to prevent seizures and, consequently, brain damage. Finding the cause of hypernatremia is essential in the treatment and prevention of recurrence. Although prenatal ultrasound and newborn screening may be useful in early diagnosis, negative results do not exclude brain and endocrine abnormalities, and further investigation may be warranted. Irrespective of the presentation, it is important to evaluate all three components (i.e., cranial midline, optic nerve, and pituitary) in patients with suspected SOD. We recommend the use of MRI in ONH patients to rule out pituitary and brain malformations. Also, extensive metabolic and endocrine workup is imperative in patients with SOD. Early and long-term management of panhypopituitarism is important in preventing hypoglycemia, metabolic disorders, developmental delay, adrenal crisis, hypotension, and eventually death.
